# Radiative Flow of Powell-Eyring Magneto-Nanofluid over a Stretching Cylinder with Chemical Reaction and Double Stratification near a Stagnation Point

**DOI:** 10.1371/journal.pone.0170790

**Published:** 2017-01-27

**Authors:** Muhammad Ramzan, Muhammad Bilal, Jae Dong Chung

**Affiliations:** 1 Department of Computer Science, Bahria University, Islamabad Campus, Islamabad, 44000, Pakistan; 2 Department of Mathematics, Faculty of Computing, Capital University of Science and Technology, Islamabad, Pakistan; 3 Department of Mechanical Engineering, Sejong University, Seoul 143-747, Korea; COMSATS Institute of Information Technology, PAKISTAN

## Abstract

This exploration addresses MHD stagnation point Powell Eyring nanofluid flow with double stratification. The effects of thermal radiation and chemical reaction are added in temperature and nanoparticle concentration fields respectively. Furthermore, appropriate transformations are betrothed to obtain nonlinear differential equations from the system of partial differential equations and an analytical solution of system of coupled differential equations is obtained by means of the renowned Homotopy Analysis method. Through graphical illustrations, momentum, energy and concentration distributions are conversed for different prominent parameters. Comparison in limiting case is also part of present study to validate the obtained results. It is witnessed that nanoparticle concentration is diminishing function of chemical reaction parameter. Moreover, mounting values of thermal and solutal stratification lowers the temperature and concentration fields respectively.

## 1 Introduction

Interest of scientists and researchers towards non-Newtonian fluids has immensely increased during the last few decades due to extensive role of these fluids in industrial and engineering applications. Examples of non-Newtonian fluids may include sugar solution, soaps, emulsions, shampoos, apple sauce, paints, cheese, muds, different cosmetic products, asphalt and ice cream etc. Since many non-Newtonian fluids with numerous characteristics are present in nature, subsequently different mathematical models are proposed to represent these fluids. The equations of different non-Newtonian fluid models are more complex and challenging in comparison to Navier-Stokes equations. That is why mathematical modelling and solutions of these fluids’ equations are of great importance. The development in mathematical modelling of non-Newtonian fluids is of great interest even today [1–6]. Eyring Powell’s model has a key role in many chemical engineering processes and has advantages over other non-Newtonian fluid models due its simplicity and ease in computations. This fluid model is obtained from Kinetic theory of liquids instead of empirical equation. Moreover, for high and low shear stresses, it decorously converts to Newtonian fluids [7]. A reasonable number of communications can be quoted highlighting Eyring–Powell fluid model in the existing literature. Akbar et al. [8] examined Eyring Powell magneto fluid flow numerically past a stretching sheet using finite difference method. Hayat et al. [9] discussed Eyring Powell fluid flow past a stretched cylinder in attendance of magneto hydrodynamic and Newtonian heating. Hayat et al. [10] investigated mixed convective stagnation point Powell Eyring fluid flow with the impact of heat generation/absorption, thermal radiation and Newtonian heating. Hayat et al. [11] studied mixed convective Eyring Powell flow with effects of Soret and Dufour. The whole scenario is deliberated in attendance of convective heat and mass boundary conditions over an exponential stretched surface. Khan et al. [12] presented numerical and analytical solution of MHD Powell Eyring fluid flow with effects of joule heating, thermophoresis and chemical reaction.

The problem of low thermal conductivity of fluids like ethylene glycol, engine oil or water is solved by the introduction of nanofluids. Excellent heat transfer features of Nanofluids as compared to ordinary base fluids have made them more beneficial to many engineering and technological processes. Applications of nanofluids include heat exchangers, cancer therapy, thermal engineering, bio medicine and thermal engineering etc. Furthermore, in physics, medicine and engineering, abundant applications related to magnetic field may be found. Magnetic field with electrically conducting fluid has many applications, *e*.*g*., boundary layer control, pumps, bearings and MHD generators. Flow behavior highly depends on intensity of applied magnetic field and orientation of fluid molecules. Moreover, suspended particles of the fluid are rearranged by the applied magnetic field. This will ultimately change the concentration of the fluid with heat transfer characteristics. A fluid possessing characteristics of liquid and magnetic properties is known as magnetic nanofluid. Abundant applications of magnetic nanofluids, e. g., optical switches, nonlinear optical materials, magneto-optical wavelength filters and optical modulators etc. can be cited in this regard. All such interesting applications have motivated scientists and researchers to look for more avenues in the field of nanofluid flows and magnetic properties [13–20].

Many researchers are attracted to Stratification because of its important role in heat and mass transfer. Stratification occurs with fluids having varied densities and in flow fields with concentration variances and differences in temperature. Practically, it is imperative to examine the impact of double stratification whenever heat and mass transfer arise collectively. In the case of lakes and ponds, stratification is imperative to keep balance in the ratio of hydrogen and oxygen so that the growth rate of species is not disturbed. Moreover, in Solar engineering too, stratification plays a vital role in obtaining enhanced energy efficiency. Many researches have been conducted in this area. To name a few, Hayat et al. [21] examined series solution of time dependent nanofluid flow with viscous dissipation, double stratification and thermal radiation effects. Abbasi et al. [22] studied Maxwell nanofluid flow with effects of mixed convection, double stratification and magnetohydrodynamic. Hayat et al. [23] explored Jeffrey fluid flow under the influence of double stratification, heat generation/absorption and mixed convection past an inclined stretched cylinder. Hayat et al. [24] found series solution of Oldroyd-B fluid flow with double stratification and chemical reaction. Kaladhar et al. [25] investigated flow of couple stress fluid with mixed convection and double stratification using Keller box method.

Motivation from the above discussion, it is of paramount interest in this article to inspect the impact of double stratification, thermal radiation and magnetohydrodynamic on Powell-Eyring nanofluid flow past a stretched cylinder. None of the above quoted references simultaneously analyzed all such effects even in viscous fluid past a stretching cylinder near a stagnation point. Homotopy Analysis method [26–30] is engaged in order to find the analytical solution of the problem.

## 2 Mathematical formulation

**Fig 1:**
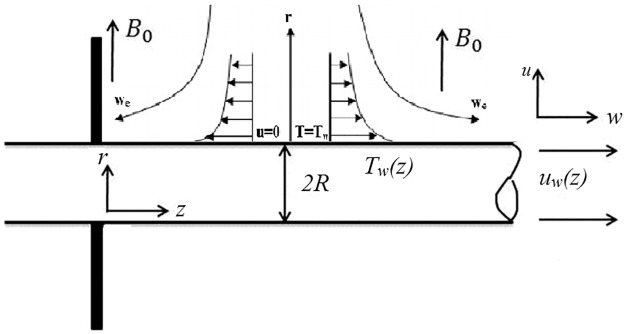
Flow diagram.

We assume a situation in which Eyring Powell nanofluid flows past a stretching cylinder with impact of double stratification, thermal radiation and magnetohydrodynamic. This study also considers chemical reaction near a stagnation point. Moreover, cylindrical coordinates with *z*–axis are along the stretched cylinder whereas *r*–axis upright to it as shown in Fig 1 Ref. [31]. In an Eyring Powell fluid [32], we have

$$\tau_{ij}=\mu \frac{\partial w_{i}}{\partial z_{j}}+\frac{1}{\beta }\sinh^{-1}\left(\frac{1}{\lambda }\frac{\partial w_{i}}{\partial z_{j}}\right)$$ (1)

The equations representing this assumed system are given by

$$\frac{\partial \left(ru\right)}{\partial r}+\frac{\partial \left(rw\right)}{\partial z}=0,$$ (2)

$$\begin{array}{ccc} u\frac{\partial w}{\partial r}+w\frac{\partial w}{\partial z} & = & w_{e}\frac{dw_{e}}{dz}+\nu \left(\frac{\partial^{2}w}{\partial r^{2}}+\frac{1}{r}\frac{\partial w}{\partial r}\right)+\frac{1}{\rho \beta C_{1}}\left(\frac{\partial^{2}w}{\partial r^{2}}+\frac{1}{r}\frac{\partial w}{\partial r}\right)-\\ & & \frac{1}{6\rho \beta C_{1}^{3}}\left(\frac{1}{r}{\left(\frac{\partial w}{\partial r}\right)}^{3}+3{\left(\frac{\partial w}{\partial r}\right)}^{2}\left(\frac{\partial^{2}w}{\partial r^{2}}\right)\right)-\frac{\sigma B_{o}^{2}\left(w-w_{e}\right)}{\rho }, \end{array}$$ (3)

$$\begin{array}{ccc} u\frac{\partial T}{\partial r}+w\frac{\partial T}{\partial z} & = & \frac{k}{\rho c_{p}}\left(\frac{\partial^{2}T}{\partial r^{2}}+\frac{1}{r}\frac{\partial T}{\partial r}\right)-\frac{1}{\rho C_{p}r}\frac{\partial }{\partial r}\left(rq_{r}\right)\\ & & \tau \left[D_{B}\frac{\partial T}{\partial r}\frac{\partial C}{\partial r}+\frac{D_{T}}{T_{\infty }}{\left(\frac{\partial T}{\partial r}\right)}^{2}\right], \end{array}$$ (4)

$$u\frac{\partial C}{\partial r}+w\frac{\partial C}{\partial z}=D_{B}\frac{1}{r}\frac{\partial }{\partial r}\left(r\frac{\partial C}{\partial r}\right)+\frac{D_{T}}{T_{\infty }}\frac{1}{r}\frac{\partial }{\partial r}\left(r\frac{\partial T}{\partial r}\right)-R_{r}\left(C-C_{\infty }\right),$$ (5)

with the boundary conditions

$$w=\frac{U_{0}z}{l},\;u=0,\;T=T_{w}=T_{0}+a\left(\frac{z}{l}\right),\;C=C_{w}\;=C_{0}+c\left(\frac{z}{l}\right)\;\;\;\text{at}\;\;r=R,$$

$$w\to w_{e}=\frac{V_{0}z}{l},\;\;\;T\to T_{\infty }=T_{0}+b\left(\frac{z}{l}\right),\;C\;\to C_{\infty }=C_{0}+d\left(\frac{z}{l}\right)\;\;\;\;\text{as}\;r\to \infty ,$$ (6)

where *u*, *w* are velocity components in *r* and *z* directions respectively. Moreover, *β* and *c*, *b* and *d*, *U*_0_, *l*, *ν*, *ρ*, *c*_*p*_, *k*, *T*, *T*_∞_, *w*_*e*_, *D*_*B*_ and *D*_*T*_ are fluid parameters, dimensionless constants, reference velocity, characteristic length, kinematic viscosity, density, specific heat, thermal conductivity, fluid temperature, ambient temperature, stretching velocity, Brownian diffusion coefficient and thermophoretic diffusion coefficient respectively.

Here, *q*_*r*_ is Rosseland radiative heat flux and is given by

$$q_{r}=-\frac{4\sigma^{*}}{3k^{*}}\frac{\partial T^{4}}{\partial z},$$ (7)

using value of *q*_*r*_ in Eq (4), we get

$$\begin{array}{ccc} u\frac{\partial T}{\partial r}+w\frac{\partial T}{\partial z} & = & \frac{k}{\rho c_{p}}\left(\frac{\partial^{2}T}{\partial r^{2}}+\frac{1}{r}\frac{\partial T}{\partial r}\right)+\frac{1}{\rho C_{p}r}\frac{4\sigma^{*}}{3k^{*}}\frac{\partial }{\partial r}\left(r\frac{\partial T^{4}}{\partial r}\right)\\ & & \tau \left[D_{B}\frac{\partial T}{\partial r}\frac{\partial C}{\partial r}+\frac{D_{T}}{T_{\infty }}{\left(\frac{\partial T}{\partial r}\right)}^{2}\right], \end{array}$$ (8)

Using the transformations of the form [33]

$$\begin{array}{ccc} \eta & = & \sqrt{\frac{U_{0}}{\nu l}}\left(\frac{r^{2}-R^{2}}{2R}\right),\;\;\;\;\;\;\;\psi =\sqrt{\frac{\nu U_{0}}{l}}Rzf\left(\eta \right),\;\phi \left(\eta \right)=\frac{C-C_{\infty }}{C_{w}-C_{\infty }}\\ w & = & \frac{U_{0}z}{l}f^{\prime }\left(\eta \right),\;u=-\sqrt{\frac{\nu U_{0}}{l}}\frac{R}{r}f\left(\eta \right),\;\;\;\;\;\theta \left(\eta \right)=\frac{T-T_{\infty }}{T_{w}-T_{\infty }}. \end{array}$$ (9)

Requirement of Eq (2), is automatically fulfilled, however, Eqs (3), (5), (6) and (8) take the form given below

$$\begin{array}{c} \left(1+2\gamma \eta \right)\left(1+M\right)f^{\prime \prime \prime }+ff^{\prime \prime }-{\left(f^{\prime }\right)}^{2}+2\gamma \left(1+M\right)f^{\prime \prime }-\frac{4}{3}\left(1+2\gamma \eta \right)M\gamma \lambda f^{\prime \prime 3}\\ -{\left(1+2\gamma \eta \right)}^{2}\lambda Mf^{\prime \prime 2}f^{\prime \prime \prime }-Ha^{2}\left(f^{\prime }-P_{0}\right)+P_{0}^{2}=0, \end{array}$$ (10)

$$\begin{array}{c} \left(1+2\gamma \eta \right)\left(1+\frac{4}{3}Rd\right)\theta^{\prime \prime }+2\gamma \left(1+\frac{4}{3}Rd\right)\theta^{\prime }+Pr\left(f\theta^{\prime }-f^{\prime }\theta -f^{\prime }e\right)+\\ PrNb\left(1+2\gamma \eta \right)\left(\theta^{\prime }\phi^{\prime }+\frac{Nt}{Nb}\theta^{\prime 2}\right)=0, \end{array}$$ (11)

$$\left(1+2\gamma \eta \right)\left(\phi^{\prime \prime }+\frac{Nt}{Nb}\theta^{\prime \prime }\right)+2\gamma \left(\phi^{\prime }+\frac{Nt}{Nb}\theta^{\prime }\right)+PrLe\left[f\phi^{\prime }-f^{\prime }\phi -f^{\prime }j\right]-Q_{0}\phi =0,$$ (12)

$$\begin{array}{ccc} f(0) & = & 0,\;\;f^{\prime }\left(0\right)=1,\;\;\theta \left(0\right)=1-e,\;\;\phi \left(0\right)=1-j,\\ f^{\prime }\left(\infty \right) & \to & P_{0},\;\;\;\theta \left(\infty \right)\to 0,\;\phi \left(\infty \right)\to 0. \end{array}$$ (13)

Here, *γ*, *M*, and λ, *Le*, *Pr*, *Ha*, *Q*_0_, *Nb*, *P*_0_ and *Nt* are curvature parameter, fluid parameters, Lewis number, Prandtl number, Hartmann number, chemical reaction parameter, Brownian motion, velocity ratio and thermophoresis parameter respectively and are given by followings:

$$\begin{array}{ccc} \gamma & = & {\left(\frac{\nu l}{U_{0}R^{2}}\right)}^{\frac{1}{2}},\;Pr=\frac{\mu c_{p}}{k},\;\;Le=\frac{\alpha }{D_{B}},\;Ha=\frac{\sigma B_{o}^{2}l}{\rho U_{0}},\\ M & = & \frac{1}{\mu \beta C_{1}},\;\;\lambda =\frac{U_{0}^{3}z^{2}}{2l^{3}C_{1}^{2}\nu },\;P_{0}=\frac{V_{0}}{U_{0}},\;\;Rd=\frac{4\sigma^{*}T_{\infty }^{3}}{kk^{*}},\;\;e=\frac{b}{a},\\ \;\;Nb & = & \frac{\tau D_{B}\left(C_{W}-C_{\infty }\right)}{\upsilon },\;\;Nt=\frac{\tau D_{T}\left(T_{W}-T_{\infty }\right)}{\upsilon T_{\infty }},\;\;j=\frac{d}{c},\;\;Q_{0}=\frac{R_{r}l}{U_{0}}. \end{array}$$ (14)

The relations of Skin friction, local Nusselt and Sherwood numbers are as follows:

$$C_{f}=\frac{\tau_{rz}}{\rho w_{e}^{2}},\;\;\;Nu=\frac{zq_{w}}{k(T_{w}-T_{\infty })},\;Sh=\;\frac{zj_{w}}{k(C_{w}-C_{\infty })}\;,\;\;$$ (15)

$$\tau_{w}={\left[\left(\mu +\frac{1}{\beta c}\right)\left(\frac{\partial w}{\partial r}\right)-\frac{1}{6\beta C_{1}^{3}}{\left(\frac{\partial w}{\partial r}\right)}^{3}\right]}_{r=R},q_{w}=-k{\left(\frac{\partial T}{\partial r}\right)}_{r=R}+{\left(q_{r}\right)}_{r=R},\;j_{w}=-{\left(\frac{\partial C}{\partial r}\right)}_{r=R},$$

and in non dimensional form

$$\begin{array}{ccc} C_{f}{Re}_{z}^{1/2} & = & \left(1+M\right)f^{\prime \prime }\left(0\right)-\frac{\lambda }{3}Mf^{\prime \prime^{3}}\left(0\right),\;\;\;\;\;Nu{Re}^{-1/2}=-\left(1+\frac{4}{3}Rd\right)\theta^{\prime }\left(0\right),\\ Sh{Re}^{-1/2} & = & -\phi^{\prime }\left(0\right), \end{array}$$ (16)

where *Re*_*z*_ = *w*_*e*_ *z*/*ν* is the Reynolds number.

## 3 Homotopic solutions

Homotopy analysis method necessitates initial guesstimates (*f*_0_, *θ*_0_, *ϕ*_0_) with auxiliary linear operators $\left(L_{f},L_{\theta },L_{\phi }\right)$ in the forms

$$\begin{array}{ccc} f_{0}\left(\eta \right) & = & P_{0}\eta +\left(1-P_{0}\right)\left(1-\exp\left(-\eta \right)\right),\;\;\;\\ \;\theta_{0}\left(\eta \right) & = & \left(1-e\right)\exp\left(-\eta \right),\;\phi_{0}\left(\eta \right)=\left(1-j\right)\exp\left(-\eta \right), \end{array}$$ (17)

$$L_{f}\left(\eta \right)=\frac{d^{3}f}{d\eta^{3}}-\frac{df}{d\eta },\;\;\;\;L_{\theta }\left(\eta \right)=\frac{d^{2}\theta }{d\eta^{2}}-\theta ,L_{\phi }\left(\eta \right)=\frac{d^{2}\phi }{d\eta^{2}}-\phi .$$ (18)

The auxiliary linear operators have the following properties

$$L_{f}\left[A_{1}+A_{2}\exp\left(\eta \right)+A_{3}\exp\left(-\eta \right)\right]=0,$$ (19)

$$L_{\theta }\left[A_{4}\exp\left(\eta \right)+A_{5}\exp\left(-\eta \right)\right]=0,$$ (20)

$$L_{\phi }\left[A_{6}\exp\left(\eta \right)+A_{7}\exp\left(-\eta \right)\right]=0,$$ (21)

where *A*_*i*_ (*i* = 1 − 7) are the arbitrary constants. Through boundary conditions, the values of these constants are given by the equations

$$\begin{array}{ccc} A_{2} & = & A_{4}=A_{6}=0,\;\;\;\;\;\;A_{3}={\left.\frac{\partial f_{m}^{⋆}\left(\eta \right)}{\partial \eta }\right|}_{\eta =0}\text{,}\;\;\;\;\;\;A_{1}=-A_{3}-f_{m}^{⋆}\left(0\right)\text{,}\;\;\;\;\;\;\;\\ A_{5} & = & -\theta_{m}^{*}\left(0\right),\;A_{7}=-\phi_{m}^{*}\left(0\right). \end{array}$$ (22)

### 3.1 Convergence analysis

**Fig 2:**
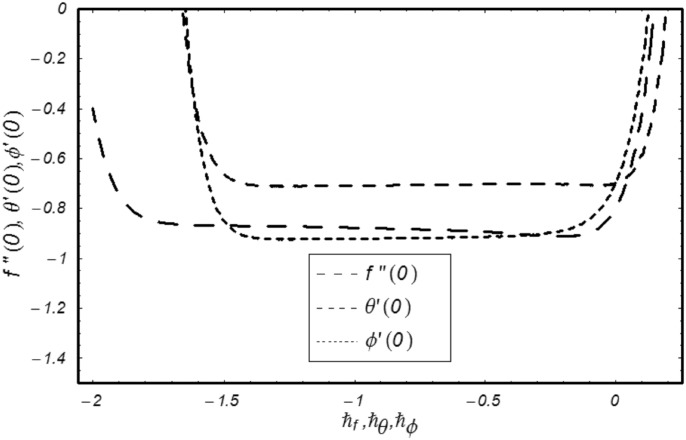
ℏ curves of *f*″(0), *θ*′(0), *ϕ*′(0).

HAM provides us with an opening to comfortably regulate and control the series solutions’ convergence. The selection of auxiliary parameters, ℏ_*f*_, ℏ_*θ*_ and ℏ_*ϕ*_ are central in regulating the convergence of desired solutions. To select suitable values of these auxiliary parameters, ℏ–curves are drawn to 14^th^ order of estimates. Fig 2 depicts the endurable values of these parameters −1.6 ≤ ℏ_*f*_ ≤ −0.3, −1.1 ≤ ℏ_*θ*_ ≤ −0.3 and −1.3 ≤ ℏ_*ϕ*_ ≤ −0.4. To validate our results obtained in Fig 2, numerical approximations to 25^th^ order of estimates as given in Table 1, are also calculated and found in good agreement.

**Table 1:** Convergence of series solutions for different order of approximations when, *M* = 0.2, *γ* = 0.3, *Ha* = 0.2, *P*_0_ = 0.2, *Q*_0_ = 0.2, *Rd* = 0.3, λ = 0.2, *J* = 0.3, *e* = 0.3, *Nt* = 0.4, *Nb* = 0.6, *Le* = 1.0, and *Pr* = 1.0.

Order of approximations	−*f*″(0)	−*θ*′(0)	−*ϕ*′(0)
1	0.86392	0.70881	0.76889
5	0.89486	0.70474	0.87450
10	0.92546	0.70257	0.90333
15	0.92925	0.70104	0.91300
20	0.93900	0.70152	0.91448
25	0.93903	0.70156	0.91563
30	0.93903	0.70156	0.91563

## 4 Results and Discussion

**Fig 3:**
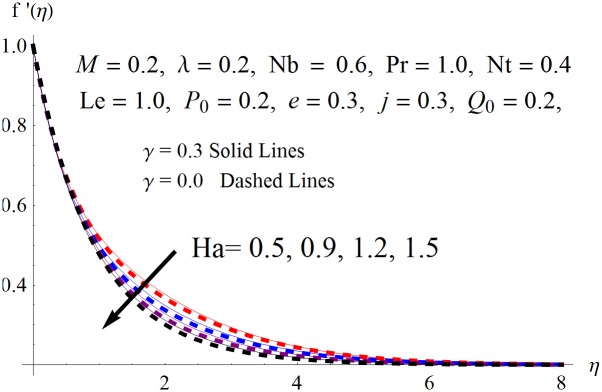
Influence of *Ha* on *f*′(*η*).

**Fig 4:**
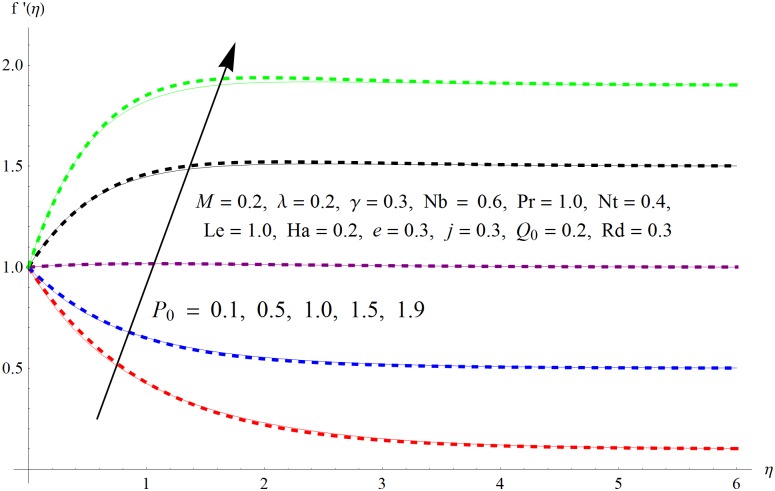
Influence of *P*_0_ on *f*′(*η*).

**Fig 5:**
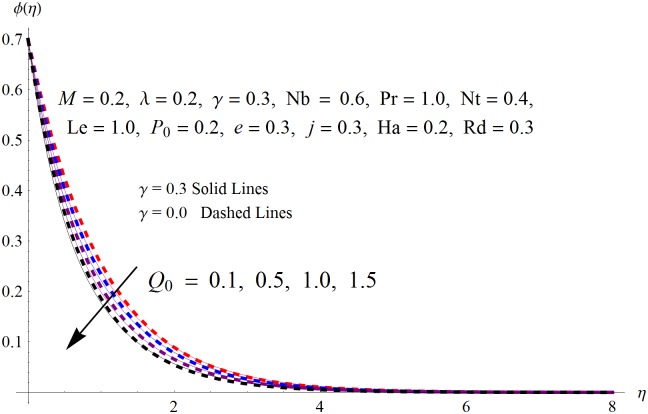
Influence of *Q*_0_ on *ϕ*(*η*).

**Fig 6:**
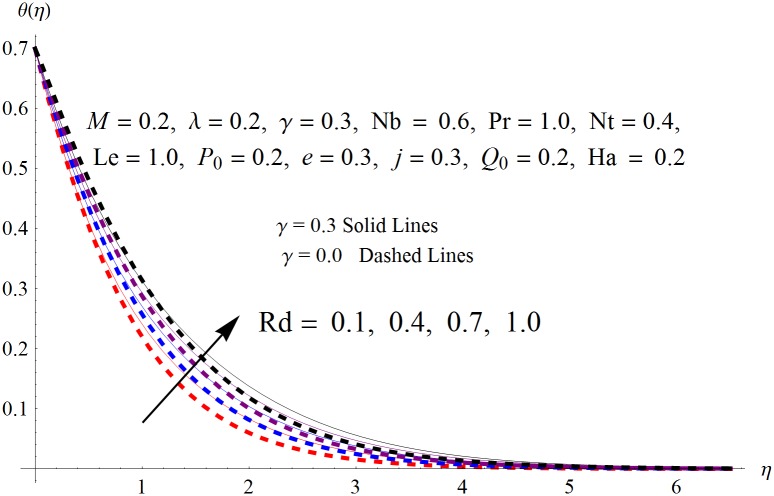
Influence of *Rd* on *θ*(*η*).

**Fig 7:**
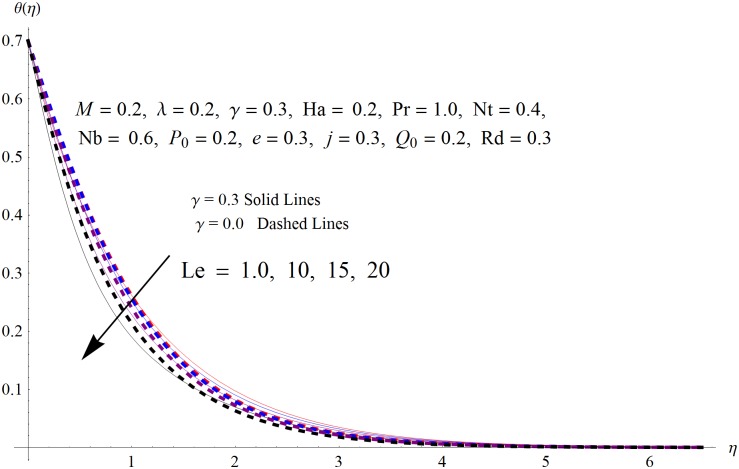
Influence of *Le* on *θ*(*η*).

**Fig 8:**
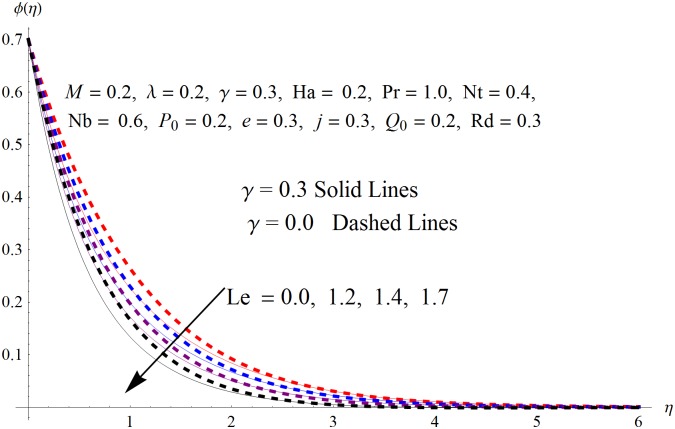
Influence of *Le* on *ϕ*(*η*).

**Fig 9:**
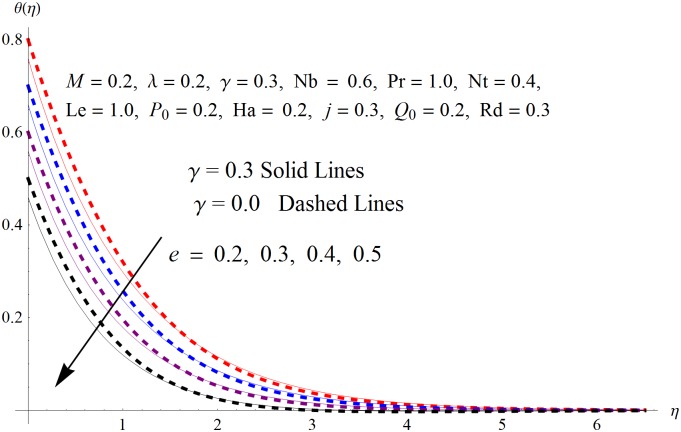
Influence of *e* on *θ*(*η*).

**Fig 10:**
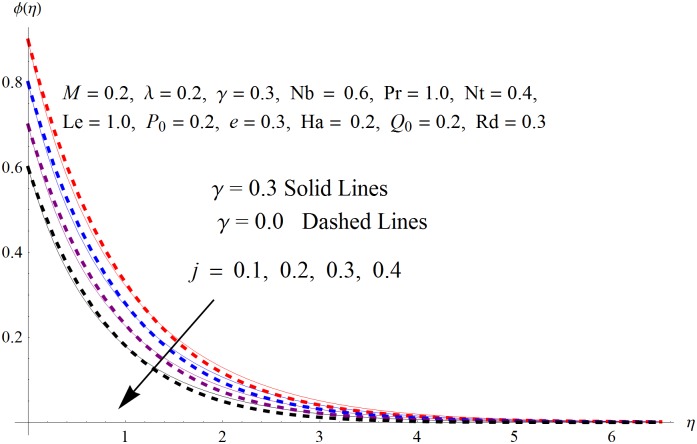
Influence of *j* on *ϕ*(*η*).

**Fig 11:**
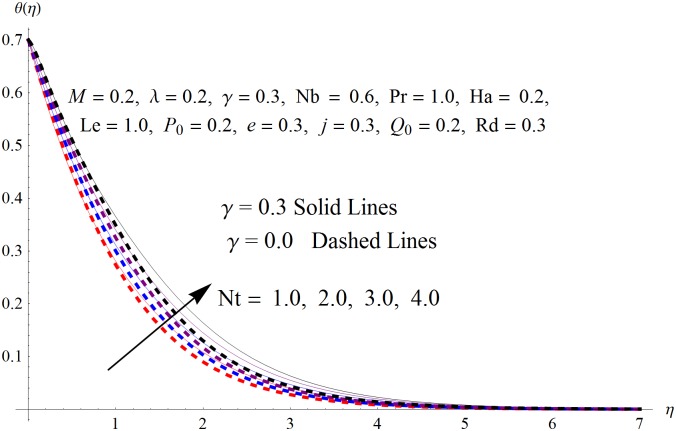
Influence of *Nt* on *θ*(*η*).

**Fig 12:**
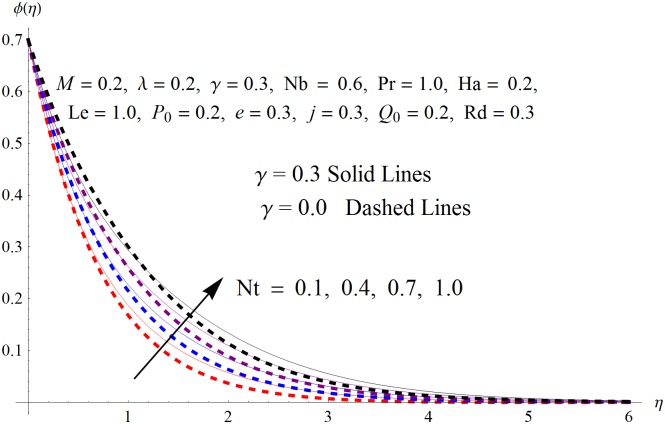
Influence of *Nt* on *ϕ*(*η*).

**Fig 13:**
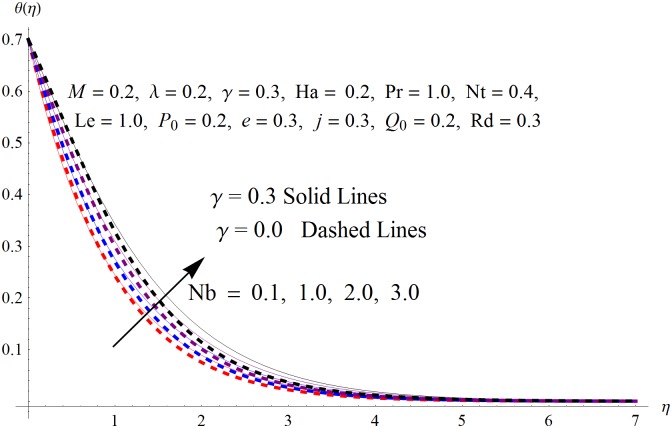
Influence of *Nb* on *θ*(*η*).

**Fig 14:**
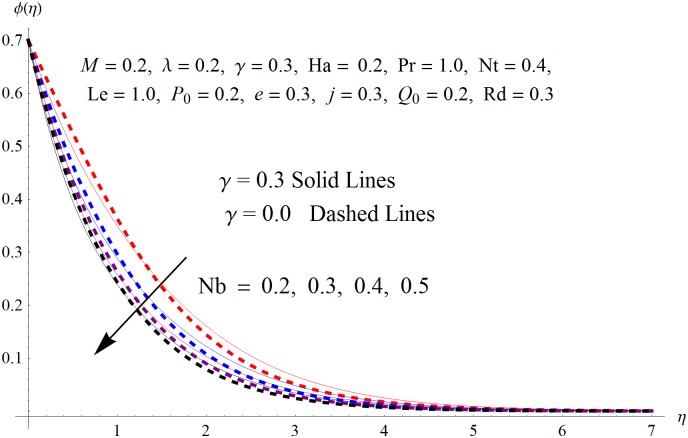
Influence of *Nb* on *ϕ*(*η*).

**Fig 15:**
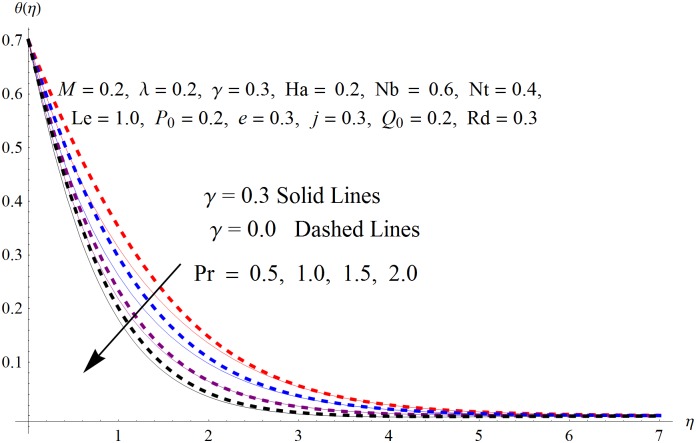
Influence of *Pr* on *θ*(*η*).

**Fig 16:**
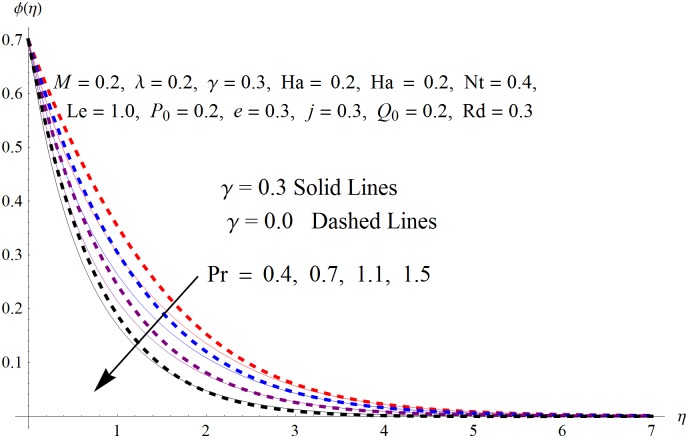
Influence of *Pr* on *ϕ*(*η*).

**Fig 17:**
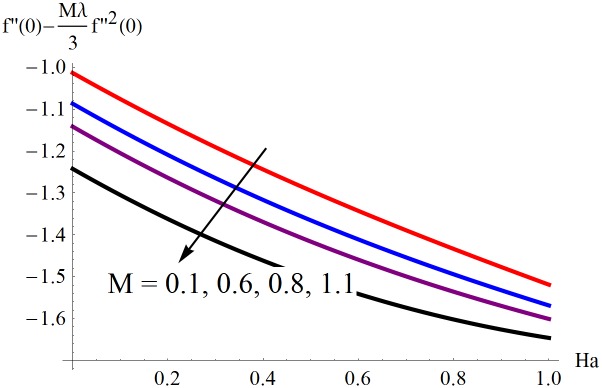
Influence of *Ha* and *M* on *C*_*f*_ *Re*^1/2^.

**Fig 18:**
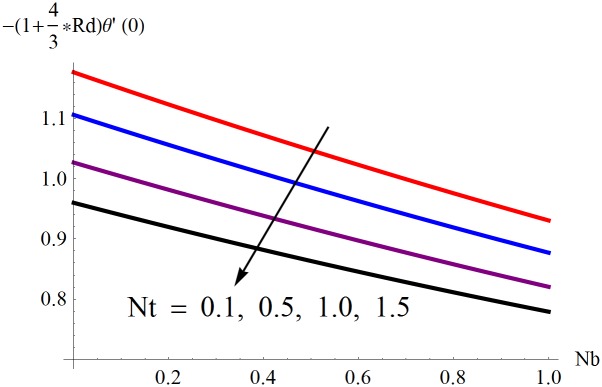
Influence of *Nt* and *Nb* on −*θ*′(0).

**Fig 19:**
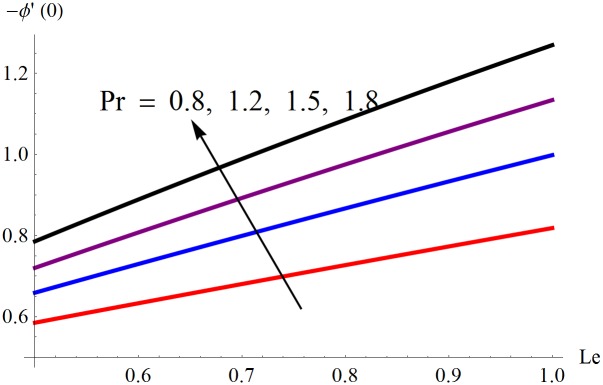
Influence of *Pr* and *Le* on −*ϕ*′(0).

**Fig 20:**
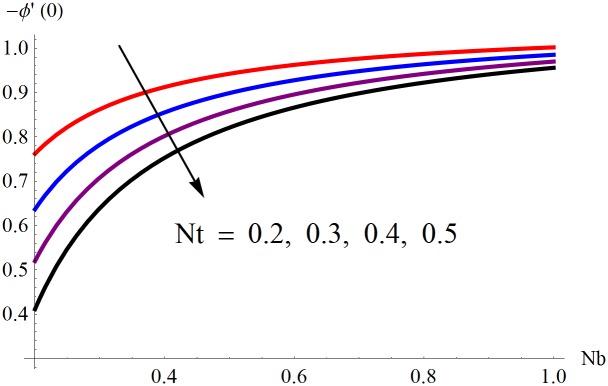
Influence of *Nt* and *Nb* on −*ϕ*′(0).

The results obtained for velocity, temperature and concentration distributions are presented graphically for prominent arising physical parameters. A comparison of flow behavior past a stretched cylinder versus a flat plat is also portrayed. Moreover, graphical illustrations depicting impact of prominent parameters on skin friction, local Nusselt and Sherwood numbers are also added to the present exploration. From Fig 3, it is perceived that the velocity profile is diminishing function of Hartmann number *Ha*. This upsurge in Hartmann number results in increase in Lorentz force. Lorentz force is a resistive force therefore attenuation in velocity profile is perceived. Furthermore, it is observed that impact of magnetic parameter is weaker in plate as compared to cylinder. Fig 4 illustrates that velocity distribution results in the mounting function of stagnation point parameter *P*_0_ in both cases when *P*_0_ < 1 and *P*_0_ > 1. This is because of the fact that cylinder’s stretching velocity is much smaller in comparison to free stream velocity. It is also noted that there is no boundary layer for *P*_0_ = 1 due to the fact that both fluid and cylinder are moving with similar velocity. Fig 5 portrays the concentration field for varied values of chemical reaction parameter *Q*_0_. Reduction in solute nanoparticle concentration and its allied boundary layer thickness is seen because of heavy disturbance in fluid’s molecules. The influence of radiation parameter *Rd* on temperature profile is examined in Fig 6. It shows that an increase in radiative parameter enhances the temperature distribution. Actually, more heat is transferred to the fluid due to higher values of radiation parameter. Moreover, it is observed that radiation effects are stronger in case of the cylinder as compared to the plate. Figs 7 and 8 depict the impact of Lewis number *Le* on temperature and nanoparticle concentration fields respectively and show that both profiles are decreasing functions of *Le*. Eventually, in the examination, a thin concentration boundary layer with frail molecular diffusivity is observed. Fig 9 shows that with gradual growth in thermal stratification *e*, temperature distribution also show a tendency to decline. This is because of temperature differences between sheet and ambient fluid which lowers the temperature field. The same fact holds in case of solutal stratification *j* and can be observed in Fig 10 where concentration profile is also the decreasing function of solutal stratification. The effects of thermophoresis parameter *Nt* on temperature and nanoparticle concentration are illustrated in Figs 11 and 12 respectively and it is perceived that both distributions are mounting functions of *Nt*. Due to increasing values of *Nt*, more nanoparticles are pulled towards the cold surface from the hot one which ultimately results in increasing the temperature and concentration distributions. Higher values of Brownian motion parameter *Nb* results in an upsurge in the temperature field but a decrease in the nanoparticle concentration profile. Both the effects are depicted in Figs 13 and 14 respectively. In fact, gradual growth in *Nb* increases the random motion and collision among nanoparticles of the fluid which produces more heat and eventually results in an increase in temperature distribution and decrease in concentration field. Figs 15 and 16 are drawn to bear witness to the variation in temperature and solutal concentration distributions for the mounting values of Prandtl number *Pr*. From these illustrations it can be seen that increasing values of *Pr* results in reducing the temperature and concentration profiles. As Prandtl number is the quotient of momentum diffusivity to thermal diffusivity. Therefore, larger values of *Pr* result because of smaller thermal diffusivity which ultimately lowers both temperature and concentration fields. Fig 17 represent the influence of Hartmann number *Ha* and fluid parameter *M* on skin friction coefficient. It is clear from the figure that skin friction coefficient is increasing function of both *Ha* and *M*. The effects of thermophoresis *Nt* and Brownian motion *Nb* parameters on local Nusselt number are displayed in Fig 18. It is detected that increasing the values of *Nt* and *Nb*, results in lowering local Nusselt number. Fig 19 is plotted to show the impact of Prandtl *Pr* and Lewis *Le* numbers on Sherwood number. Here, it is noted that Sherwood number is mounting function of both *Pr* and *Le*. However, from Fig 20, it is clear that Sherwood number is increasing and decreasing function of *Nb* and *Nt* respectively.

In Table 2, a comparison to the previous exploration in the limiting case for the Sherwood number is presented. All obtained results are found in excellent agreement.

**Table 2:** Comparison of values of Sherwood number in limiting case for varied values of *M*, λ, *Ha*, *γ*, *Pr*, *Nt*, *Nb*, *Rd* and *Le* when *P*_0_ = *e* = *j* = *Q*_0_ = 0.

*M*	λ	*Ha*	*γ*	*Pr*	*Nt*	*Nb*	*Rd*	*Le*	Hayat et al. [34]	Present
0.2	0.1	0.2	0.1	1.2	0.1	0.1	0.1	1.3	0.6658	0.6658
0.4									0.6849	0.6849
0.6									0.7008	0.7008
0.2	0.1								0.6658	0.6658
	0.5								0.6641	0.6641
	0.7								0.6633	0.6633
	0.1	0.2							0.6658	0.6658
		0.5							0.6377	0.6377
		0.7							0.6080	0.6080
		0.2	0.1						0.6658	0.6658
			0.3						0.7497	0.7497
			0.5						0.8318	0.8318
			0.1	0.7					0.8657	0.8657
				1.2					0.6658	0.6658
				1.7					0.4896	0.4896
				1.2	0.10				0.6658	0.6658
					0.20				0.1311	0.1311
					0.22				0.0279	0.0279
					0.1	0.1			0.6658	0.6658
						0.3			0.7869	0.7869
						0.5			1.140	1.140
							0.1		0.6658	0.6658
							0.4		0.7869	0.7869
							0.7		0.8671	0.8671
								0.9	0.3601	0.3601
								1.3	0.6658	0.6658
								1.7	0.9193	0.9193

## 5 Final remarks

The present study explores the effects of double stratification and magnetohydrodynamic flow of Eyring Powell nanofluid past a stretched cylinder near stagnation point. The analysis is done in the presence of chemical reaction and thermal radiation. Homotopy analysis method is used in order to obtain the series solutions of the said problem. The salient features of the present study are as following.

- Increasing the values of chemical reaction parameter results in reducing the solute nanoparticle concentration.
- Velocity profile decreases when radiation parameter increases.
- Sherwood number is increasing and decreasing function of *Nb* and *Nt* respectively.
- Gradual growth in thermal and solutal stratification results in decline in the temperature and concentration distributions respectively.
- Higher values of Brownian motion parameter Nb increase the temperature field but decrease the nanoparticle concentration profile.

## References

[1] Ramzan M., Farooq M., Hayat T., Chung J. D., Radiative and Joule heating effects in the MHD flow of a micropolar fluid with partial slip and convective boundary condition, Journal of Molecular Liquids 221 (2016) 394–400. 10.1016/j.molliq.2016.05.091

[2] Ramzan M., Influence of Newtonian heating on three dimensional MHD flow of couple stress nanofluid with viscous dissipation and joule heating, PLoS ONE, 10(4) (2015) e0124699 10.1371/journal.pone.0124699 25874800 PMC4397014

[3] Ramzan M., Bilal M., Three-dimensional flow of an elastico-viscous nanofluid with chemical reaction and magnetic field effects, Journal of Molecular Liquids, 215 (2016) 212–220. 10.1016/j.molliq.2015.12.036

[4] Abbas Z., Sheikh M., Motsa S. S., Numerical solution of binary chemical reaction on stagnation point flow of Casson fluid over a stretching/shrinking sheet with thermal radiation, Energy, 95 (2016) 12–20. 10.1016/j.energy.2015.11.039

[5] Hayat T., Jabeen S., Shafiq A., Alsaedi A., Radiative squeezing flow of second grade fluid with convective boundary conditions, PLoS ONE 11(4) (2015) e0152555 10.1371/journal.pone.0152555 27096616 PMC4838271

[6] Hayat T., Makhdoom S., Awais M., Saleem S., Rashidi M. M., Axisymmetric Powell-Eyring fluid flow with convective boundary condition: optimal analysis, Applied Mathematics and Mechanics, 37(7) (2016) 919–928. 10.1007/s10483-016-2093-9

[7] Powell R. E., Eyring H. Mechanism for relaxation theory of viscosity, Nature, 154 (1944) 427–428. 10.1038/154427a0

[8] Akbar N. S., Ebaid A., Khan Z. H., Numerical analysis of magnetic field effects on Eyring-Powell fluid flow towards a stretching sheet, Journal of Magnetism and Magnetic Materials, 382 (2015) 355–358.

[9] Hayat T., Hussain Z., Farooq M., Alsaedi A., MHD flow of Powell-Eyring fluid by a stretching cylinder with newtonian heating, Thermal Science, (2016) 10.1371/journal.pone.0156955 PMC4900525 27280883

[10] Hayat T., Waqas M., Shehzad S. A., Alsaedi A., Mixed Convection Stagnation-Point Flow of Powell-Eyring Fluid with Newtonian Heating, Thermal Radiation, and Heat Generation/Absorption, Journal of Aerospace Engineering, (2016) 1943–5525. 10.1061/(ASCE)AS.1943-5525.0000674

[11] Hayat T., Saeed Y., Alsaedi A., Asad S., Effects of convective heat and mass transfer in flow of Powell-Eyring fluid past an exponentially stretching sheet, PLoS ONE, 10(9) (2015) e0133831 10.1371/journal.pone.0133831 26327398 PMC4556520

[12] Khan N. A., Sultan F., Khan N. A., Heat and mass transfer of thermophoretic MHD flow of Powell–Eyring fluid over a vertical stretching sheet in the presence of chemical reaction and joule heating, International Journal of Chemical Reactor Engineering, 13(1) (2015) 37–49.

[13] Hussanan A, Salleh M. Z., Khan I., Tahar R. M. and Ismail Z., Soret effects on unsteady magnetohydrodynamic mixed-convection heat-and-mass-transfer flow in a porous medium with Newtonian heating, Maejo International Journal of Science and Technology, 9(2) (2015) 224–245.

[14] Hussanan A, Ismail Z., Khan I., Hussein A. G., Shafie S., Unsteady boundary layer MHD free convection flow in a porous medium with constant mass diffusion and Newtonian heating, The European Physical Journal Plus, 129 (2014) 46 10.1140/epjp/i2014-14046-x

[15] Hussanan A., Khan I., Hashim H., Anuar M. K., Ishak N., Md. Sarif N., Salleh M. Z., Unsteady MHD flow of some nanofluids past an accelerated vertical plate embedded in a porous medium, Jurnal Teknologi, 78(2) (2016) 121–126. 10.11113/jt.v78.4900

[16] Ska M. T., Das K., Kundu P. K., Effect of magnetic field on slip flow of nanofluid induced by a non-linear permeable stretching surface, Applied Thermal Engineering, 104 (2016) 758–766.

[17] Hayat T., Aziz A., Muhammad T., Ahmad B., Influence of magnetic field in three-dimensional flow of couple stress nanofluid over a nonlinearly stretching surface with convective condition, PLoS ONE, 10(12) (2015) e0145332 10.1371/journal.pone.0145332 26714259 PMC4694771

[18] Ramzan M., Bilal M., Three-dimensional flow of an elastico-viscous nanofluid with chemical reaction and magnetic field effects, Journal of Molecular Liquids, 215 (2016) 212–220. 10.1016/j.molliq.2015.12.036

[19] Hussain T., Shehzad S. A., Hayat T., Alsaedi A., Solamy F. A., Ramzan M., Radiative hydromagnetic flow of Jeffrey nanofluid by an exponentially stretching sheet, PLoS, ONE, 9(8) (2014) e103719 10.1371/journal.pone.0103719 25084096 PMC4118940

[20] Dogonchi A. S., Ganji D. D., Investigation of MHD nanofluid flow and heat transfer in a stretching/shrinking convergent/divergent channel considering thermal radiation, Journal of Molecular Liquids, 220 (2016) 592–603. 10.1016/j.molliq.2016.05.022

[21] Hayat T., Imtiaz M., Alsaedi A., Unsteady flow of nanofluid with double stratification and magnetohydrodynamics, International Journal of Heat and Mass Transfer, 92 (2016) 100–109. 10.1016/j.ijheatmasstransfer.2015.08.013

[22] Abbasi F. M., Shehzad S. A., Hayat T., Ahmad B., Doubly stratified mixed convection flow of Maxwell nanofluid with heat generation/absorption, Journal of Magnetism and Magnetic Materials, 404 (2016) 159–165. 10.1016/j.jmmm.2015.11.090

[23] Hayat T., Qayyum S., Farooq M., Alsaedi A., Mixed convection flow of Jeffrey fluid along an inclined stretching cylinder with double stratification effect, Thermal Science, (2015) 52–52. 10.2298/TSCI141106052H

[24] Hayat T., Muhammad T., Shehzad S. A., Alsaedi A., Temperature and concentration stratification effects in mixed convection flow of an Oldroyd-B fluid with thermal radiation and chemical reaction, PLoS ONE, 10(6) (2015) e0127646 10.1371/journal.pone.0127646 26102200 PMC4478041

[25] Kaladhar K., Double stratification effects on mixed convection flow of couple stress fluid in a non-Darcy porous medium with heat and mass fluxes, Computational and Applied Mathematics, (2015) 1–16. 10.1007/s40314-015-0248-x

[26] Ramzan M., Inam S., Shehzad S. A., Three dimensional boundary layer flow of a viscoelastic nanofluid with Soret and Dufour effects, Alexandria Engineering Journal, 55 (2016) 311–319. 10.1016/j.aej.2015.09.012

[27] Liao S. J., On the homotopy analysis method for nonlinear problems, Applied Mathematics and Computation, 147 (2004) 499–513. 10.1016/S0096-3003(02)00790-7

[28] Ramzan M., Yousaf F., Boundary layer flow of three-dimensional viscoelastic nanofluid past a bi-directional stretching sheet with Newtonian heating, AIP Advances, 5 (2015) 057132 10.1063/1.4921312

[29] Shehzad S. A., Hussain T., Hayat T., Ramzan M., Alsaedi A., Boundary layer flow of third grade nanofluid with Newtonian heating and viscous dissipation, Journal of Central South University, 22 (2015) 360–367. 10.1007/s11771-015-2530-x

[30] Ramzan M., Farooq M., Hayat T., Alsaedi A., Cao J., MHD stagnation point flow by a permeable stretching cylinder with Soret-Dufour effects, Journal of Central South University, 22 (2015) 707–716. 10.1007/s11771-015-2574-y

[31] Qasim M., Khan Z. H., Khan W. A., Shah I. A., MHD boundary layer slip flow and heat transfer of Ferrofluid along a stretching cylinder with prescribed heat flux, PLoS ONE, 9(1) (2014) e83930 10.1371/journal.pone.0083930 24465388 PMC3898924

[32] Qasim M., Soret and Dufour effects on the flow of an Erying-Powell fluid over a flat plate with convective boundary condition, Eur. Phys. J. Plus 129: (2014) 24 10.1140/epjp/i2014-14024-4

[33] Hayat T., Hussain Z., Alsaedi A., Farooq M., Magnetohydrodynamic flow by a stretching cylinder with Newtonian heating and homogeneous-heterogeneous reactions, PLoS ONE, 11(6) (2016) e0156955 10.1371/journal.pone.0156955 27280883 PMC4900525

[34] Hayat T., Gull N., Farooq M., Ahmad B., Thermal radiation effect in MHD flow of Powell Eyring nanofluid induced by a stretching cylinder, Journal of Aerospace Engineering, 29(1) (2016). 10.1061/(ASCE)AS.1943-5525.0000501

